# A genetically encoded biosensor for visualising hypoxia responses *in vivo*

**DOI:** 10.1242/bio.018226

**Published:** 2016-12-23

**Authors:** Tvisha Misra, Martin Baccino-Calace, Felix Meyenhofer, David Rodriguez-Crespo, Hatice Akarsu, Ricardo Armenta-Calderón, Thomas A. Gorr, Christian Frei, Rafael Cantera, Boris Egger, Stefan Luschnig

**Affiliations:** 1 Institute of Molecular Life Sciences and Ph.D. program in Molecular Life Sciences, University of Zurich, Zurich CH-8057, Switzerland; 2 Developmental Neurobiology, IIBCE, Montevideo 116 00, Uruguay; 3 Department of Biology, University of Fribourg, Fribourg CH-1700, Switzerland; 4 LS Instruments AG, Fribourg CH-1700, Switzerland; 5 Institute of Veterinary Physiology, University of Zurich, Zurich CH-8057, Switzerland; 6 Institute of Cell Biology, Swiss Federal Institute of Technology, Zurich CH-8093, Switzerland; 7 Zoology Department, Stockholm University, Stockholm 106 91, Sweden; 8 Institute of Neurobiology, University of Münster, Badestrasse 9, Münster D-48149, Germany; 9 Cells-in-Motion Cluster of Excellence (EXC 1003–CiM), University of Münster, Münster D-48149, Germany

**Keywords:** Hypoxia, HIF-1, Prolyl hydroxylase, Biosensor, Tracheal system, *Drosophila*

## Abstract

Cells experience different oxygen concentrations depending on location, organismal developmental stage, and physiological or pathological conditions. Responses to reduced oxygen levels (hypoxia) rely on the conserved hypoxia-inducible factor 1 (HIF-1). Understanding the developmental and tissue-specific responses to changing oxygen levels has been limited by the lack of adequate tools for monitoring HIF-1 *in vivo.* To visualise and analyse HIF-1 dynamics in *Drosophila*, we used a hypoxia biosensor consisting of GFP fused to the oxygen-dependent degradation domain (ODD) of the HIF-1 homologue Sima. GFP-ODD responds to changing oxygen levels and to genetic manipulations of the hypoxia pathway, reflecting oxygen-dependent regulation of HIF-1 at the single-cell level. Ratiometric imaging of GFP-ODD and a red-fluorescent reference protein reveals tissue-specific differences in the cellular hypoxic status at ambient normoxia. Strikingly, cells in the larval brain show distinct hypoxic states that correlate with the distribution and relative densities of respiratory tubes. We present a set of genetic and image analysis tools that enable new approaches to map hypoxic microenvironments, to probe effects of perturbations on hypoxic signalling, and to identify new regulators of the hypoxia response.

## Abstract (precis)

**Summary:** This study describes a biosensor for visualising the hypoxic state of cells *in vivo*. They demonstrate that the *Drosophila* larval brain contains distinct hypoxic microenvironments that correlate with local airway supply.

## INTRODUCTION

Cells adapt to oxygen (O_2_) deprivation (hypoxia) by triggering a hypoxia response, which adjusts metabolism to low levels of O_2_. The hypoxia response pathway is evolutionarily conserved and has crucial roles for cell survival under challenging physiological, environmental, and pathological conditions, including stroke, ischemia and cancer (reviewed in Semenza, 2014). The master regulator of the hypoxia response is the hypoxia inducible factor (HIF), a heterodimeric transcription factor consisting of the regulatory HIF-alpha subunit (Wang and Semenza, 1995) and the constitutive HIF-beta subunit, both of which contain basic helix-loop-helix (bHLH) and PER-ARNT-SIM (PAS) domains (Wang et al*.*, 1995). The HIF-alpha/HIF-beta heterodimer binds to hypoxia response elements (HREs; reviewed in Wenger et al*.*, 2005) and activates the expression of hypoxia response genes, including those encoding erythropoietin, glucose transporters, glycolytic enzymes, and vascular endothelial growth factor (VEGF) (Manalo et al*.*, 2005; Schödel et al*.*, 2011). Under normoxia, prolyl hydroxylase domain (PHD) 2-oxoglutarate-dependent dioxygenases hydroxylate HIF-alpha on specific proline residues in the oxygen-dependent degradation domain (ODD) (Epstein et al*.*, 2001; Huang et al*.*, 1998; Ivan et al*.*, 2001; Jaakkola et al*.*, 2001). Prolyl-hydroxylated HIF-alpha is recognised by the von Hippel Lindau (VHL) protein, the substrate recognition subunit of an E3 ubiquitin ligase (Iwai et al*.*, 1999; Lisztwan et al*.*, 1999), which targets HIF-alpha for proteasomal degradation (Maxwell et al*.*, 1999). As PHD uses 2-oxoglutarate and molecular oxygen as co-substrates, prolyl hydroxylase activity directly depends on the partial pressure of oxygen (pO_2_), rendering PHD enzymes bona fide O_2_ sensors (Ivan et al*.*, 2001). In addition to prolyl hydroxylation, mammalian HIF-1, but not *Drosophila* HIF-1, is also hydroxylated in an O_2_-dependent manner on asparagine by the asparaginyl hydroxylase FIH-1 (factor inhibiting HIF-1), which prevents the transcriptional activation of target genes by HIF-1 under high O_2_ partial pressures (Lando et al*.*, 2002).

Although HIF was originally identified as a regulator of responses to changing O_2_ levels, it is now known to control a wide variety of functions, including hormonal regulation, energy metabolism, cell migration, growth, apoptosis, angiogenic signalling and matrix and barrier functions (reviewed in Schofield and Ratcliffe, 2004). Mammalian tumour growth and resistance to various therapies was shown to be dependent upon HIF, making components of the HIF pathway promising drug targets (Eltzschig et al*.*, 2014). However, most tissues also experience varying O_2_ concentrations during normal development, and different organs and tissues are exposed to a wide range of O_2_ concentrations. Stem cells reside in hypoxic niches (Eliasson and Jönsson, 2010; Mohyeldin et al*.*, 2010), and Notch signalling has been shown to maintain stem cell niches in a HIF-dependent manner (Gustafsson et al*.*, 2005). Thus far, the HIF pathway has been mainly studied biochemically at the level of hypoxia-induced degradation and nucleo-cytoplasmic transport of HIF-alpha protein, as well as at the level of the transcriptional response regulated by HIF-1 (Manalo et al*.*, 2005; Schödel et al*.*, 2011). However, studying the regulation of HIF signalling during development and under changing environmental conditions has been limited by the lack of adequate tools to monitor HIF signalling *in vivo*.

Given the conservation of the HIF pathway, *Drosophila melanogaster* provides a powerful model system to address this issue. The bHLH/PAS domain transcription factor Similar (Sima) is the only known HIF-alpha homolog in *Drosophila* (Bacon et al*.*, 1998). Under normoxia the prolyl hydroxylase Fatiga (Fga) hydroxylates a single proline residue (P850; Arquier et al*.*, 2006) in the ODD of Sima (Lavista-Llanos et al*.*, 2002). Hydroxylated Sima is targeted for degradation by the dVHL ubiquitin ligase (Irisarri et al*.*, 2009). Under hypoxia, non-hydroxylated Sima accumulates in the cytosol and translocates to the nucleus (Irisarri et al*.*, 2009), where it dimerises with the HIF-beta subunit Tango (Tgo; Lavista-Llanos et al*.*, 2002), binds to HREs, and regulates transcription of hypoxia response genes.

Here we present a fluorescent protein biosensor that reproduces the response of endogenous Sima protein to changing O_2_ concentrations. The ratiometric design of the sensor allows visualisation in living animals with high sensitivity of the relative levels of HIF accumulation, a key step in the hypoxia response. We use the sensor to demonstrate that the cellular hypoxic status varies between different tissues and between cells within a tissue at ambient normoxia. As in other insects, oxygen is supplied to most tissues in *Drosophila* by a network of tracheal tubes (Ghabrial et al*.*, 2003), the growth and branching of which is promoted by hypoxia (Centanin et al*.*, 2010; Gorr et al*.*, 2006; Jarecki et al*.*, 1999; Wigglesworth, 1954). Gas exchange takes place between the thinnest tracheal tubes (tracheoles) and the cells in their proximity. However, owing to the lack of suitable tools, a correlation between the extent of tracheal supply and the hypoxic state of cells has not been demonstrated directly thus far. We used the hypoxia biosensor to show that the cellular hypoxic status in the larval brain correlates with the extent of tracheolation in different brain regions. We present a set of genetic and image analysis tools that enable new approaches for mapping hypoxic microenvironments, to probe the effects of perturbations on hypoxic signalling, and to identify new regulators of the hypoxia response *in vivo*.

## RESULTS

### Construction of a genetically encoded biosensor to visualise hypoxia responses *in vivo*

**Fig. 1.**
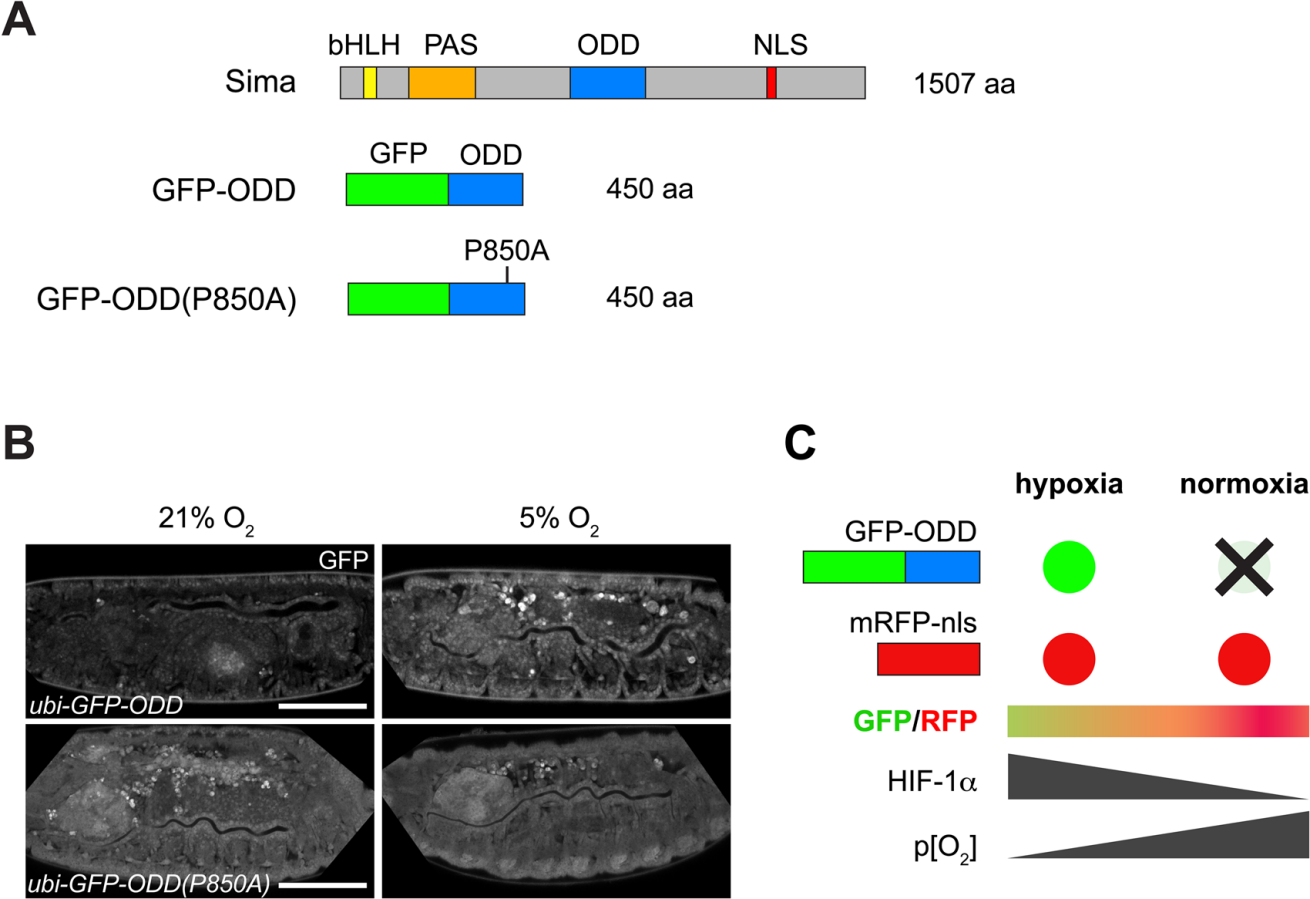
**Construction of an oxygen-sensitive fluorescent protein-based biosensor.** (A) Schematic representations of full-length Sima protein (dHIF1-alpha; top), with its O_2_-dependent degradation domain (ODD; aa 673-895), of the GFP-ODD fusion protein (middle), and of the GFP-ODD construct with the P850 residue mutated into alanine [GFP-ODD(P850A); bottom]. (B) Embryos showing the sensitivity of GFP-ODD to O_2_ tension. GFP-ODD signals (top panels) were reduced in embryos incubated at 21% O_2_ compared to embryos incubated at 5% O_2_. The P850A mutation renders the GFP-ODD construct largely insensitive to changing O_2_ levels (bottom panels). Scale bar: 100 µm. (C) Schematic representation of the ratiometric sensor design. The red fluorescence from a control protein (mRFP-nls) remains relatively constant under changing O_2_ concentrations, whereas GFP-ODD is sensitive to O_2_ levels, providing a measure of the cellular hypoxia response state.

To construct a sensor that reflects the O_2_-dependent variation of Sima levels, we fused the oxygen-dependent degradation domain (ODD; aa 692-863; Lavista-Llanos et al*.*, 2002) of Sima to the C-terminus of green fluorescent protein (GFP-ODD) and placed this construct under the control of the *ubiquitin-69E* (*ubi*) promoter for constitutive expression throughout development (Fig. 1A). Under normoxia (21% ambient O_2_), GFP-ODD was distributed in the cytoplasm and nuclei of embryonic (Fig. 1B; Irisarri et al*.*, 2009) and larval cells, as well as in transiently transfected S2R+ cells (data not shown). GFP-ODD signals were lower in embryos kept under normoxia (21% O_2_) compared to embryos kept under hypoxia (5% O_2_; Fig. 1B) for 17 h prior to imaging, suggesting that GFP-ODD accumulates in hypoxic cells. Since the O_2_-dependent degradation of Sima requires hydroxylation of proline 850 (P850) in the ODD (Arquier et al*.*, 2006; Centanin et al*.*, 2008; Irisarri et al*.*, 2009), we also generated a GFP-ODD version with P850 mutated into alanine (P850A; Fig. 1A) as a control. As expected, O_2_-dependent degradation of GFP-ODD required the presence of P850 (Fig. 1B, Fig. S1) (Arquier et al*.*, 2006; Centanin et al*.*, 2008; Irisarri et al*.*, 2009). These findings suggest that GFP-ODD reflects the behaviour of full-length Sima protein under changing O_2_ concentrations. However, besides regulating the stability of the GFP-ODD protein, changing O_2_ concentrations influence gene expression in *Drosophila* (Azad et al*.*, 2009; Liu et al*.*, 2006), as well as maturation of the GFP fluorophore (Coralli et al*.*, 2001; Heim et al*.*, 1994). Thus, in order to detect even subtle O_2_-dependent changes in GFP-ODD levels, and to exclude confounding effects of changing O_2_ conditions on gene expression and on fluorophore maturation, we co-expressed along with GFP-ODD a second protein whose stability is not influenced by changing O_2_ levels, but which is otherwise subject to the same environmental differences. For this goal we used monomeric red fluorescent protein with a nuclear localisation signal (mRFP-nls) under the control of the *ubi* promoter to normalise *ubi*-GFP-ODD signal intensity. O_2_-dependent changes in gene expression (transcription, translation) should equally affect *ubi*-GFP-ODD and *ubi*-mRFP-nls levels. Thus, at a given O_2_ concentration and in any cell type, changes in the ratio between GFP-ODD and mRFP-nls signals should only depend on the O_2_-triggered degradation of GFP-ODD (Fig. 1C). Hence, this ratio should provide a measure for Sima degradation independent of absolute protein levels and of cell type-specific expression of the hypoxia sensor.

### GFP-ODD responds to changes in the ambient oxygen concentration

**Fig. 2.**
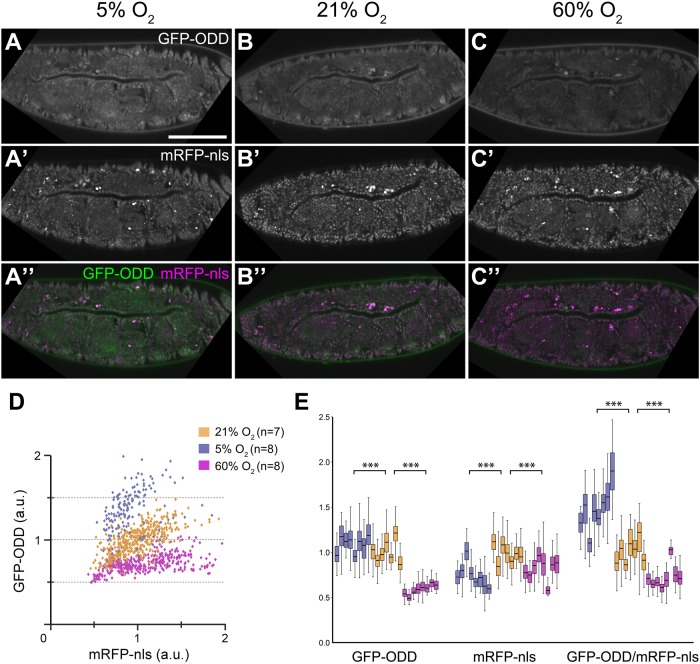
**GFP-ODD responds to changes in ambient O_2_ concentrations.** Embryos incubated at three different O_2_ concentrations show decreasing levels of GFP-ODD fluorescence with the highest levels under hypoxia (5% O_2_; A), lower levels under normoxia (21% O_2_; B) and further reduced levels under hyperoxia (60% O_2_; C). (A′,B′,C′) Conversely, fluorescence intensity of mRFP-nls shows comparatively little changes in the three O_2_ conditions. (A″,B″,C″) The merge of the two channels, indicating the GFP-ODD (green)/mRFP-nls (magenta) ratio, as basis for the ratiometric analysis. (D) Analysis of the hypoxic state of individual cells. GFP-ODD and mRFP-nls intensities were analysed in nuclei of embryonic tracheal cells. The scatter plot shows normalised intensities (a.u., arbitrary units) of each channel, representing changes in fluorescence signals. Each point corresponds to a single nucleus. The number of embryos (n) analysed for each condition is indicated. (E) Changes in individual fluorescence channels and GFP-ODD/mRFP-nls ratios for each O_2_ condition. Each box plot represents data from a single embryo in which fluorescence was measured in at least 35 cells. Scale bar: 100 µm. ***P≤0.001; Mann-Whitney U-test.

To test whether GFP-ODD responds to different O_2_ concentrations we exposed embryos (collected within one hour after egg lay; 0-1 h AEL) to 5, 21 or 60% O_2_ for 17 h, and subsequently measured the GFP and mRFP-nls signals in tracheal and epidermal cells (Fig. 2 and data not shown). Embryos developed into viable larvae at 5% and 60% O_2_. In embryos kept at 5% O_2_ (Fig. 2A) the levels of GFP-ODD were elevated compared to embryos reared under normoxia (Fig. 2B), whereas embryos reared at 60% O_2_ showed reduced GFP-ODD levels (Fig. 2C). mRFP levels decreased slightly under both 5% and 60% O_2_ (Fig. 2A′-C′ and Fig. 2E), probably reflecting changes in maturation or stability of the mRFP fluorophore (Cecic et al*.*, 2007). Overall, two-colour imaging revealed increased GFP-ODD/mRFP-nls intensity ratios with decreasing O_2_ concentration (Fig. 2A″-C″). Measurements of GFP-ODD and mRFP-nls fluorescence intensities in individual tracheal cell nuclei showed a clear separation between the three O_2_ conditions (Fig. 2D). Notably, GFP-ODD/mRFP-nls ratios varied between different embryos kept at the same O_2_ level, and from cell to cell within individual embryos. Interestingly, this variation was larger under hypoxia than under normoxia. These findings indicate that the ratiometric hypoxia sensor is capable of detecting changes in reporter accumulation at the level of individual cells.

### Modulation of hypoxia pathway components results in changes in GFP-ODD levels

**Fig. 3.**
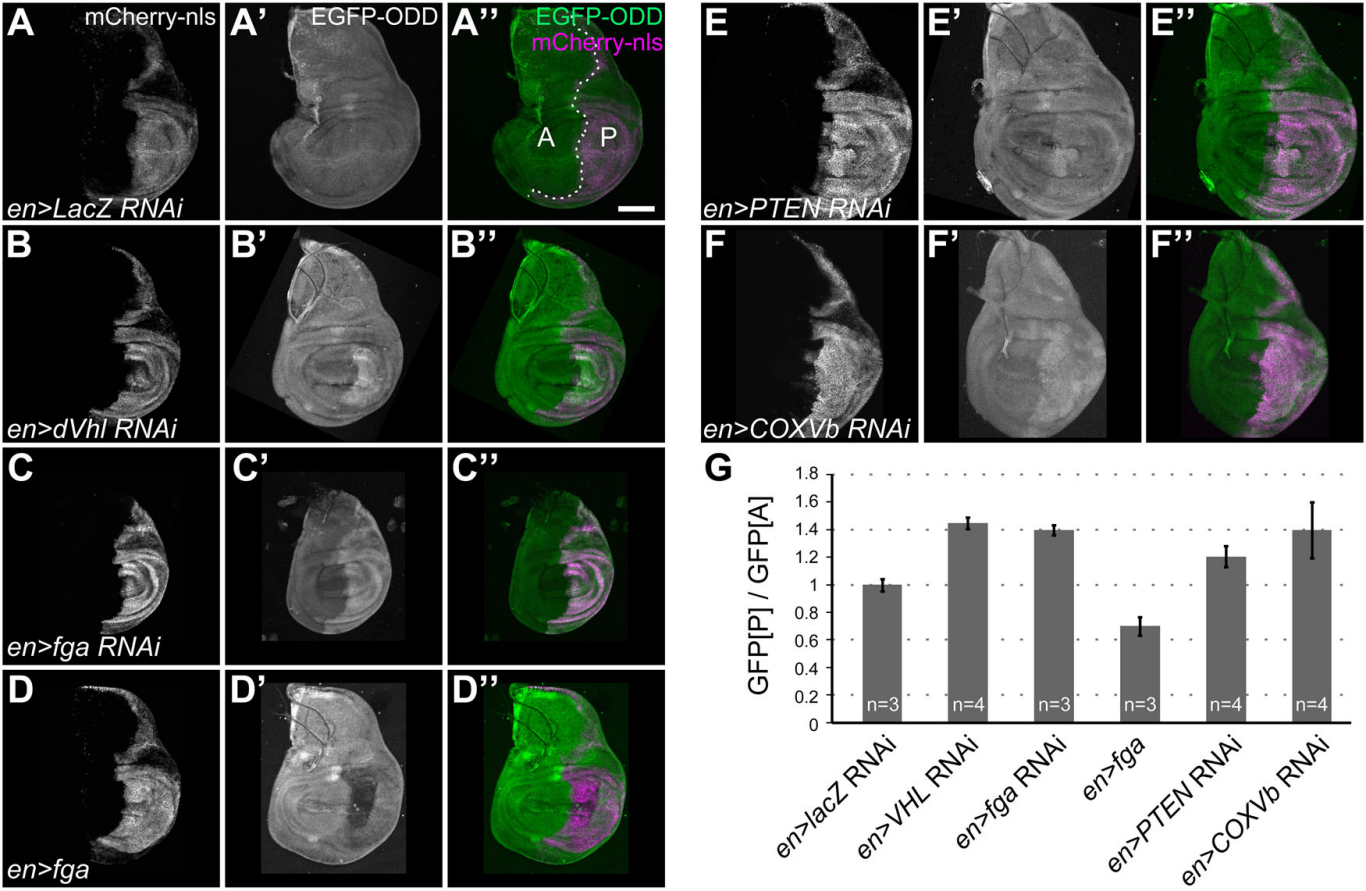
**GFP-ODD responds to modulation of HIF pathway components.** Levels of GFP-ODD fluorescence (green) were analysed in wing imaginal discs of third-instar larvae, in which regulators of the hypoxia response were either depleted by RNAi or overexpressed in the posterior compartment using the *en*-Gal4 driver. Cells in the posterior compartment (marked ‘P’ in panel A″) are labelled by the *en*-Gal4-driven expression of UAS-mCherry-nls (A, magenta in A″). The anterior compartment (marked ‘A’ in panel A″) serves as an internal control. In control larvae (lacZ-RNAi; A-A″), GFP-ODD levels in the anterior and posterior compartment are indistinguishable. RNAi-mediated knockdown of *dVHL* (B-B″) or *fatiga* (C-C″) in the posterior compartment results in accumulation of GFP-ODD. Conversely, overexpression of Fatiga-A leads to lower levels of GFP-ODD (D-D″). (E-E″) Depletion of PTEN in the posterior compartment results in accumulation of GFP-ODD. (F-F″) Inhibition of the mitochondrial respiratory chain through COXVb RNAi causes accumulation of GFP-ODD. Scale bar: 100 µm. (G) Bar graph showing ratios between mean GFP-ODD intensities in the posterior (GFP[P]) and anterior compartment (GFP[A]). Ratios were normalised to the values of the RNAi control (*en*>lacZ RNAi). Bars indicate mean values, error bars represent the standard deviation. The number (n) of imaginal discs analysed for each genotype is indicated.

We next investigated GFP-ODD responses after perturbations of selected HIF-pathway components in the larval wing imaginal disc, an epithelial tissue with a sharp boundary between its anterior and posterior compartments and amenable to precise genetic manipulation. Although highly proliferative during larval stages, wing imaginal discs are not supplied by functional tracheoles (Jarecki et al*.*, 1999; Sato and Kornberg, 2002) and presumably rely on diffusion from the surrounding hemolymph for gas exchange. We used *engrailed-*Gal4 (*en*-Gal4)-driven UAS-RNAi constructs to deplete selected HIF pathway components specifically in the posterior compartment of the wing disc, and we compared GFP-ODD levels between the posterior (experimental, marked by mCherry-nls expression) and the anterior (control) compartment (Fig. 3). While a control RNAi (lacZ; Fig. 3A-A″) showed no effect, knockdown of *dVHL* (Fig. 3B-B″) or *fatiga* (Fig. 3C-C″) under the control of *en*-Gal4 resulted in 1.4-fold increased accumulation of GFP-ODD in the posterior compartment (Fig. 3G). Conversely, overexpression of Fga in the posterior compartment led to lower levels of GFP-ODD relative to the anterior compartment (Fig. 3D-D″,G). These results show that GFP-ODD reproduces the regulation of endogenous Sima protein by the HIF pathway.

To see if this system can be employed to discover new factors involved in the HIF response, we examined the effect of abnormal growth on HIF signalling. Loss of the tumour suppressor PTEN results in upregulation of hypoxia response genes in mammalian tumours (Zundel et al*.*, 2000). Likewise, loss of PTEN in *Drosophila* leads to overgrowth (Huang et al*.*, 1999), but it has not been tested whether a hypoxia response is also induced in this situation. Indeed, knockdown of PTEN in the posterior compartment of the wing disc led to increased accumulation of GFP-ODD, concomitant with overgrowth (Fig. 3E-E″), whereas overexpression of PTEN had no visible effect (data not shown). We also tested the effect of impaired mitochondrial respiration on the hypoxia response. Mitochondrial respiration is a major source of reactive oxygen species (ROS), which arise as by-products of the electron transport chain, and blocking the chain can lead to increased ROS production (reviewed in Murphy, 2009). ROS were proposed to influence the HIF response through inhibition of PHD by peroxide (Guzy and Schumacker, 2006). Indeed, we found that interfering with the respiration chain through RNAi-mediated depletion of the cytochrome c subunit Vb (COXVb) in the posterior compartment of wing discs led to the accumulation of GFP-ODD in that region (Fig. 3F-F″). We hypothesise that the inhibition of mitochondrial respiration triggered a hypoxia response through elevated ROS produced upon the deactivation of cytochrome c oxidase. Together, these findings underscore the applicability of our sensor for identifying new factors involved in hypoxia responses, using genetic or pharmacological screens.

### GFP-ODD accumulation correlates inversely with tracheal supply in the larval brain

**Fig. 4.**
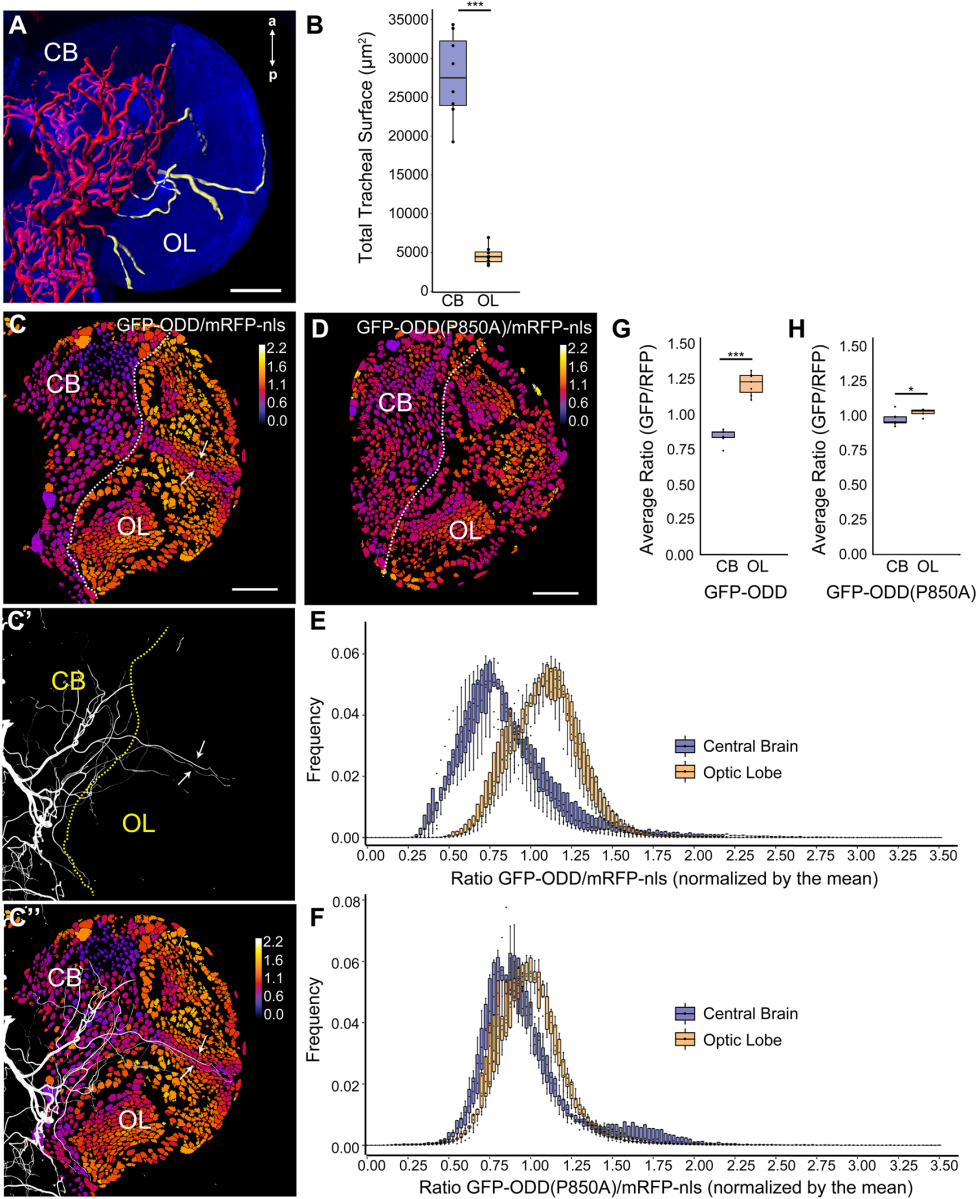
**Different tracheolation densities within the brain correlate with distinct cellular hypoxic states.** (A) 3D reconstruction of the tracheal system in a brain hemisphere of a larva (96 hALH). Maximum intensity projection of anti-Dlg antibody staining (blue) shows outline of the brain. Tracheoles are coloured red in the central brain (CB) and yellow in the optic lobe (OL). The brain's midline is to the left and anterior (a) and posterior (p) are indicated. See also Movie 1. (B) Box plot showing quantification of total tracheal surface within the CB (blue) and OL (orange) (CB=27720.1 µm^2^, OL=4647.8 µm^2^; *n*=8 brains; Mann–Whitney *U*-test, *P*=0.00015). The box plot shows maximum and minimum observation, upper and lower quartile, and median. (C) Single frontal confocal section of a brain hemisphere of a larva (96 hALH) expressing ubi-GFP-ODD and ubi-mRFP-nls. The colour code (upper right) indicates average GFP-ODD/mRFP-nls ratios for each nucleus. (C′) Maximum intensity projection of the brain's tracheal system (white). (C″) Superposition of the images shown in (C) and (C′) illustrates the topographical correlation between the differential tracheolation of CB and OL regions and their different hypoxic states. Low ratios correlate with dense tracheolation in the CB. Higher ratios correlate with the sparsely tracheolated OL. Note that cells adjacent to the OL lateral tracheoles (arrows) exhibit lower ratios, consistent with O_2_ diffusion across few cell diameters. (D) Single frontal confocal section of a brain hemisphere of a larva (96 hALH) expressing ubi-GFP-ODD(P850A) and ubi-mRFP-nls. The colour code (upper right) indicates average GFP-ODD(P850A)/mRFP-nls ratios for each nucleus. (E) Histogram representing the frequency distribution of GFP-ODD/mRFP-nls ratios for the CB and the OL, showing a clear separation, *n*=7 brain hemispheres. (F) Histogram representing the frequency distribution of GFP-ODD(P850A)/mRFP-nls ratios for CB and OL, which show a large overlap, *n*=7 brain hemispheres. Data in E and F is presented as box plots. (G) Box plots showing the average GFP-ODD/mRFP-nls ratios, calculated from the data shown in E. Note the significantly higher average ratios in the OL compared to the CB (1.11 in OL vs 0.83 in CB; student's *t*-test, *P*=1.256e-06). (H) Box plots showing the average ODD(P850A)/mRFP-nls ratios for CB and OL, calculated from the data shown in F. Note the overlap in average ratios between the non-tracheolated OL region and the CB (1.02 in OL vs 0.97 in CB; student's *t*-test, *P*=0.03). Box plots in E,F,G and H show maximum and minimum observation, upper and lower quartile, and median. Scale bars: 40 µm.

To challenge the potential of our hypoxia sensor in terms of spatial resolution, we studied the developing larval brain because it exhibits very different degrees of tracheal supply in two well-defined regions (Pereanu et al*.*, 2007). While the central brain (CB) is densely tracheolated, the optic lobes (OL), crescent-shaped structures occupying the lateral region of each hemisphere, receive only few tracheal branches (Fig. 4A,B). We generated 3D reconstructions to compare the tracheolation of the CB and OL (measured as tracheal surface) 96 h after larval hatching (hALH; Fig. 4A, Movie 1). This revealed that the total tracheal surface is six times larger in the CB than in the OL (CB=27720.1 µm^2^, OL=4647.8 µm^2^; *n*=8 brains; Mann–Whitney *U*-test, *P*=0.00015, Fig. 4B). We hypothesised that the scarce tracheolation of the OL should result in a weaker oxygenation of this region relative to the more densely tracheolated central brain. Hence, lower O_2_ levels in the OL relative to the CB should translate into reduced oxygen-dependent degradation of GFP-ODD in the OL. To test this hypothesis, we developed an image analysis pipeline based on a custom ImageJ plugin (Fig. S2) and measured the ratio of GFP-ODD and mRFP-nls mean intensities in every segmented cell nucleus of larval brain hemispheres. This analysis provided a portrayal of O_2_ distribution at the single-cell level by representing the GFP-ODD/mRFP-nls ratio in every nucleus using a heat map (Fig. 4C,C″, Movie 2). We observed striking differences between CB and OL. Whereas most CB nuclei showed low GFP-ODD/mRFP-nls ratios, presumably reflecting high oxygenation, OL nuclei generally displayed markedly higher ratios, consistent with lower oxygenation of this region (Fig. 4E,G). Strikingly, the range of GFP-ODD/mRFP-nls ratios correlated with the differential tracheolation pattern in the developing brain (Fig. 4C-C″). To quantify this topographic correlation we measured the GFP-ODD/mRFP-nls ratios on each confocal section throughout the brain and plotted the distribution of ratio frequencies (Fig. 4E; *n*=7 brains). Indeed, the histogram shows a significant shift in the frequency distribution towards higher ratios for OL nuclei as compared to CB nuclei (Fig. 4E). The average ratio was significantly higher in the OL than in the CB (1.11 in OL vs 0.83 in CB; student's *t*-test, *P*=1.256e-06; Fig. 4G). This correlation was most prominent in the inner cell plug (ICP; Hofbauer and Campos-Ortega, 1990). ICP cells, which are in close vicinity to the lateral optic lobe tracheoles that run from the CB through the inner cell plug of the OL (Pereanu et al*.*, 2007), showed lower ratios compared to cells of the neighbouring medulla cortex (Fig. 4C,C′, arrows).

To corroborate that these region-specific responses of the sensor are due to oxygen-dependent regulation of GFP-ODD protein, we imaged brains expressing the GFP-ODD(P850A) mutant and mRFP-nls (Fig. 4D, Movie 3). Whereas clear regional differences in ratiometric values were observed between CB and OL for wild-type GFP-ODD, such regional differences were not seen with GFP-ODD(P850A) (Fig. 4D). The distribution of ratios in the CB and in the OL largely overlapped (Fig. 4F) and the GFP-ODD (P850A)/mRFP-nls average ratios were similar in the OL and CB (1.02 in OL vs 0.97 in CB; student's *t*-test, *P*=0.03, *n*=7 brains; Fig. 4H). Taken together, these results validate the applicability of the hypoxia sensor as a tool to visualise tissue hypoxia *in vivo* and demonstrate that the spatial resolution provided by our method is sufficient to detect differential hypoxia responses within tissues and at the level of individual cells.

## DISCUSSION

Here we present a genetically encoded biosensor that can be used to visualise and quantify changes in the hypoxia response state with single-cell resolution throughout development in the living animal. We demonstrate that the reporter is sensitive to changes in ambient O_2_ concentrations, and that it faithfully reflects the effects of genetic manipulations (depletion or overexpression) of known regulators of HIF.

Fluorescent protein-based approaches for detecting responses to hypoxia in cultured cells or *in vivo* have been described previously (Arquier et al*.*, 2006; Fomicheva et al*.*, 2008; Harada et al*.*, 2007), but the performance of these hypoxia sensors has been limited by the intrinsic dependency of GFP and RFP on oxygen for fluorophore maturation (Cecic et al*.*, 2007; Coralli et al*.*, 2001; Heim et al*.*, 1994). A recent elegant study has circumnavigated this problem by making use of the oxygen-independent fluorescent protein UnaG as a transcriptional reporter for HIF-dependent gene expression (Erapaneedi et al*.*, 2016).

A previous study described a biosensor consisting of GFP fused to the ODD of human HIF-alpha (Arquier et al*.*, 2006). Surprisingly, in contrast to our results, Arquier et al. (2006) found no response of epidermal cells to hypoxia using the GFP-ODD(HIF-alpha) sensor. While this difference may be attributable to the human ODD sequence used by Arquier et al. (2006), we consider it more likely that the apparently greater sensitivity of our method relies on the use of a second fluorescent protein signal as an internal reference. Our ratiometric approach allows us to distinguish subtle relative differences in hypoxic status, which may escape detection by single-colour sensor systems.

Compared to other approaches, our sensor provides several specific advantages for the analysis of hypoxia responses. First, it reveals spatial information with single-cell resolution, which is lost in whole-animal transcriptomic or proteomic analyses. Second, our system detects changes directly at the level of the cellular O_2_ sensor, rather than downstream transcriptional responses, and therefore avoids the delay associated with transcriptional reporters, such as *ldh*-GAL4-UAS-GFP (Lavista-Llanos et al*.*, 2002). Transcriptional reporters get stably induced by a hypoxic episode, but do not, or only with a significant delay, report the dynamics of the response, such as equilibration after re-oxygenation. Third, our ratiometric hypoxia reporter is compatible with a variety of genetic manipulations, including mutations, RNAi, and over-expression, and thus provides a versatile tool for a wide range of approaches to examine hypoxia signalling and its relation to physiological and pathological conditions.

We noticed considerable differences when we applied the sensor to characterise the hypoxic status of cells in different tissues. Whereas embryonic epidermal and tracheal cells show varying levels of reporter accumulation, apparently reflecting the individual state of each cell at the moment of the analysis, the larval brain contains distinct regions where most cells appear to exist under significantly different degrees of hypoxia. The different hypoxic states of these regions correlate with the extent of local tracheolation. The presence of distinct microenvironments with different degrees of oxygenation is likely to be functionally important for the maintenance of a proliferative region and a differentiated region within the brain. Neural and other stem cells reside in hypoxic niches, which provide a protective environment essential for maintaining the correct balance between self-renewal and differentiation (De Filippis and Delia, 2011; Mohyeldin et al*.*, 2010). It will be interesting to investigate whether the hypoxia detected by our biosensor in the OL area is sufficient to keep stem cells in a self-renewal mode of maintenance, and whether the more dense tracheal supply in the central brain favours neuronal differentiation in this region. Analysing the effects of altered oxygenation on neural stem cell behaviour, as well as on neuronal activity and on animal behaviour, will be greatly aided by the ability to visualise and quantify hypoxia signalling using our sensor.

The terminal tracheal branching pattern of *Drosophila* larvae was proposed to reflect the history of hypoxic episodes experienced by cells of the target tissues (Jarecki et al*.*, 1999). Experiments to test this hypothesis have been limited by the lack of tools to visualise HIF accumulation *in vivo*. The sensor described here may be used to monitor the ‘hypoxia response history’ of individual cells or of regions within a given tissue. Our system may further be adapted to investigate processes regulated by oxygen physiology, such as the relationship between hypoxia and the behaviour of stem cells, tumours and blood vessels *in vivo* in other genetically and optically tractable model organisms.

## MATERIALS AND METHODS

### *Drosophila* stocks

Fly stocks are described in FlyBase (flybase.org) unless mentioned otherwise. The following UAS-RNAi lines were obtained from the Vienna *Drosophila* RNAi Center (http://stockcenter.vdrc.at): *fatiga* #103382, *dVHL* #108920, *sima* #106187, *COXVb* #30892, #105769, *PTEN* #101475. UAS-lacZ-RNAi was a gift from Peter Gallant (University of Würzburg, Germany). Other fly stocks were *en*-Gal4, UAS-mCherry-nls and UAS-Fatiga-A [gift from Pablo Wappner (Instituto Leloir, Buenos Aires, Argentina)]. For ratiometric analyses, an insertion of ubi-GFP-ODD on the second chromosome was recombined with ubi-mRFP-nls (BL#34500). For control experiments an insertion of ubi-GFP-ODD(P850A) on the second chromosome was crossed to ubi-mRFP-nls (BL#34500).

### DNA constructs and transgenes

pWRPE-pubi-GFP-ODD was generated as follows: EGFP was amplified from pUASt-nls-EGFP with ATATGGTACCCAACATGGTGAGCAAGGGCGAG (P1; KpnI, forward) and GGACAAATCGTCAGGCTTGTACAGCTCGTCCAT (P2; reverse). The Sima ODD fragment was amplified with CCTGACGATTTGTCCCACCA (P3; forward) and ATATGAATTCTTATGGTGGGCACCACATG (P4; EcoRI, reverse) from *sima* cDNA clone 5.1 (Gorr et al*.*, 2004). Purified PCR products were used for fusion PCR with P1 and P4 to generate GFP-ODD, which was cut with KpnI and EcoRI, inserted into pWRPE-pubi (kan^R^) and confirmed by sequencing.

pWRPE-pubi-GFP-ODD(P850A) was generated as follows: ODD(P850A) was synthesised (GenScript Inc., USA) with a base change corresponding to the P850A mutation and inserted into pUC57 using BamHI and HindIII restriction sites. The ODD(P850A) fragment and EGFP were amplified by PCR and subsequently joined by fusion PCR. The resulting fragment was inserted into pWRPE-pubi using KpnI and BcuI sites and confirmed by sequencing. Transgenic flies were generated using P-Element-mediated germline transformation.

### Hypoxia and hyperoxia treatment

Embryos were collected for one hour at 25°C, dechorionated in 7% sodium hypochlorite for 3 min, washed, placed on apple juice agar plates, and transferred into sealed chambers (1 litre), which were flushed with pre-mixtures of either 5% or 60% O_2_ in N_2_. After 16 h at 22°C, embryos were immediately mounted in 10S halocarbon oil and imaged within 20 min so as to minimise the effects of re-oxygenation. For treatment of larvae, eggs were collected for 3 h on apple juice agar plates supplied with yeast. Larvae were allowed to develop until early third-instar stage. Samples of 50 larvae were collected, washed, and placed on a new apple juice agar plate with a minimal amount of yeast. Plates were placed into sealed containers, which were flushed with premixed N_2_ and O_2_ every 4 h.

### Larval dissection and immunostainings

Larvae were dissected in fixative immediately after hypoxic incubations so as to minimise effects of re-oxygenation. Third-instar larval wing imaginal discs were dissected in 4% Paraformaldehyde containing Hoechst 33258. Larval brains were dissected in 4% formaldehyde in 0.1 M phosphate-buffered saline (PBS, pH 7.4), 0.5 mM EGTA, 5 mM MgCl_2_ and fixed for a total of 18 min including the dissection, washed with PBS containing 0.1% Triton-X, and prepared for immunostaining. Mouse anti-Discs Large antibody 4F3 (1:30; DSHB, Iowa, USA; Parnas et al*.*, 2001) was used to stain cell cortices. The chitinous cuticle lining of the tracheal lumen was stained using Calcofluor (1:200; Sigma-Aldrich). Secondary antibodies were conjugated to Alexa488, Alexa568, or Alexa633 (1:200; Molecular Probes).

### Microscopy

Live embryos were mounted in 10S halocarbon oil and examined using a 40× objective on an Olympus Fluoview 1000 confocal microscope. Larval brain preparations were imaged using a 40× objective on a Leica TCS SPE II confocal microscope. Optical sections through an entire brain hemisphere were recorded at 0.6 µm intervals for tracheal surface measurements or at 1 µm intervals for ratiometric analysis.

### Image analysis

Confocal images were analysed with Imaris 7.6 (Bitplane) and Fiji (Schindelin et al*.*, 2012) and assembled using Adobe Photoshop 8.0. For analyses of living embryos, nuclei were segmented on single confocal sections in the control (mRFP-nls) channel either manually (embryonic trachea) or automatically (embryonic epidermis) using the Thresholding, Watershed and Analyze Particles plugins in Fiji. Absolute intensities of EGFP and mRFP-nls were recorded within the segmented nuclei. Then the EGFP/mRFP-nls ratio was calculated for each nucleus by dividing the intensity in the green channel (mean of all pixel intensities) by the intensity in the red channel (mean of all pixel intensities). The mean of the ratios of the normoxic (21% O_2_) samples was used as the normalisation factor. The normalised ratio for a given nucleus was calculated by dividing the ratio of absolute intensities by the normalisation factor. The mean normalised ratio for an entire embryo was calculated by averaging the normalised ratios for individual nuclei of the embryo. For quantification of GFP-ODD in wing imaginal discs, regions of interest (ROIs) of the same size were selected in the anterior and posterior compartments and fluorescence intensity was recorded for each ROI. Ratios were calculated from mean GFP-ODD intensities in the anterior and posterior compartment.

Investigators were not blinded to allocation during experiments and samples were not randomised for the allocation to experimental and control groups.

#### Tracheal surface measurements

Measurements of tracheal surfaces were done separately in the OL and in the CB. The TrackEM plugin in Fiji was used to separate OL and CB following the neuroanatomical borders outlined by anti-Dlg staining. Subsequently, Imaris 7.6 was used to segment and quantify the surfaces of all tracheoles in the two regions.

#### Ratiometric image analysis in larval brains

The ratiometric analysis involves two major steps. First, ROIs are segmented using intensity threshold segmentation. Second, pixel intensities are measured in the ROI for the sensor signal (GFP-ODD) and the control signal (nuclear mRFP). The ratio of the average pixel intensities between the sensor signal and the control signal is calculated for each ROI. We wrote a macro in Fiji (Schindelin et al*.*, 2012) to facilitate and semi-automate threshold segmentation of multi-image confocal stacks. Parameters outlined in the macro, such as background subtraction, and the choice of threshold algorithm can be easily adapted to the user's sample. The resulting image mask is subsequently used to select the ROI. To carry out the ratiometric analysis, we provide a Fiji plugin, which we named Blob Ratiometric 2D. The macro is available on Github at https://github.com/eggerbo/Drosophila_brain and the plugin source code is available at https://github.com/eggerbo/ImageJ_BlobRatiometric. Detailed instructions are provided in the Readme files. A release of the plugin ready to install in Fiji is available for download at https://github.com/eggerbo/ImageJ_BlobRatiometric/releases.

The nuclear mRFP-nls signal was used to segment nuclei. Prior to segmentation, background subtraction was performed on a neighbourhood of 40 pixels using the rolling ball algorithm. To de-noise, the images were smoothened using an average filter on a 3×3 neighbourhood. To obtain ROIs corresponding to nuclei in each confocal section, autothreshold segmentation was applied (default method) and regions were subsequently separated using the Watershed algorithm. In each ROI the average pixel intensity was calculated for the reference signal (mRFP-nls) and the sensor signal (GFP). These values were used to calculate the GFP-ODD/mRFP-nls signal ratio for each nucleus. In the segmentation mask each nuclear ROI was false-coloured according to its mean ratio value using a heat-map lookup-table. Ratio values shown on images and graphs were normalised to the mean value of all nuclei for each brain hemisphere. To quantify mean ratios and the frequency distribution of different brain regions, the central brain and optic lobe regions were manually separated using TrackEM in Fiji. Graphs were prepared using R studio software.
